# The efficacy of neuromuscular electrical stimulation with alternating currents in the kilohertz frequency to stimulate gait rhythm in rats following spinal cord injury

**DOI:** 10.1186/s12938-015-0094-5

**Published:** 2015-10-29

**Authors:** Tsukasa Kanchiku, Hidenori Suzuki, Yasuaki Imajo, Yuichiro Yoshida, Atsushi Moriya, Yutaka Suetomi, Norihiro Nishida, Youhei Takahashi, Toshihiko Taguchi

**Affiliations:** Department of Orthopedic Surgery, Yamaguchi University Graduate School of Medicine, Ube, Yamaguchi 755-8505 Japan

## Abstract

**Background:**

Rehabilitation facilitates the reorganization of residual/regenerated neural pathways and is key in improving motor function following spinal cord injury. Neuromuscular electrical stimulation (NMES) has been reported as being clinically effective. Although it can be used after the acute phase post-injury, the optimal stimulation conditions to improve motor function remain unclear. In this paper, we examined the effectiveness of NMES with alternating currents in the kilohertz (kHz) frequency in gait rhythm stimulation therapy.

**Methods:**

Tests were performed using 20 mature female Fischer rats. Incomplete spinal cord injuries (T9 level) were made with an IH impactor using a force of 150 kdyn, and NMES was administered for 3 days from the 7th day post-injury. The needle electrodes were inserted percutaneously near the motor point of each muscle in conscious rats, and each muscle on the left and right leg was stimulated for 15 min at two frequencies, 75 Hz and 8 kHz, to induce a gait rhythm. Motor function was evaluated using Basso, Beattie, Bresnahan (BBB) scores and three-dimensional (3D) gait analysis. Rats were divided into four groups (5 rats/group), including the NMES treatment 75-Hz group (iSCI-NMES 75 Hz), 8-kHz group (iSCI-NMES 8 kHz), injury control group (iSCI-NT), and normal group (Normal-CT), and were compared.

**Results:**

There was no significant difference in BBB scores among the three groups. In 3D gait analysis, compared with the injury control group, the 8-kHz group showed a significant improvement in synergistic movement of both hindlimbs.

**Conclusion:**

We suggest that kHz stimulation is effective in gait rhythm stimulation using NMES.

## Background

A moderate-to-high level of spinal cord injury generally presents with poor recovery of motor function. Improvement in function is limited when therapies are used alone, and a combination of several effective treatments may be required [1]. Rehabilitation promotes the intrinsic plasticity of the nervous system, which accelerates the recovery of motor function after incomplete spinal cord injury, and it is hoped that rehabilitation will facilitate the reorganization of neural pathways after regenerative therapy [1]. Repetitive rhythmical movement of the legs facilitated by robot-assisted walking on a treadmill and combined with functional electrical stimulation therapy is clinically effective [2–4].

Neuromuscular electrical stimulation (NMES) has been used as a means of restoring limb motor function that has been lost primarily due to spinal cord injury and stroke. In experiments using monkeys, it has been found that, when part of the input/output pathway into the corticocerebral motor area is damaged, the functional map of the corticocerebral motor area undergoes plastic changes [5, 6]. In humans, studies using functional magnetic resonance imaging have shown that NMES-induced articular movement activates the brain area closer to automatic movement than that to hyperkinetic movement [7]; moreover, it has been reported that repetitive movement helps reorganize the functional map [8]. Thus, NMES is not simply a way to restore function but has also been used as a means of motor function training, the clinical effectiveness of which has been reported [3, 9–11]. However, the details of the mechanism that underlies the improvement in motor function remain unclear [12]. Basic research including animal experiments is needed to fully understand the mechanism of functional recovery and examine its effectiveness when combined with spinal cord regeneration therapies. However, there are relatively few reports on the effectiveness of rehabilitation combined with spinal cord regeneration therapies based on animal models. Therefore, this area needs to be explored further, including the development of suitable experimental animal models [1, 12].

Edgerton and Roy provided a detailed report of a treadmill gait-training animal model [13], whereas Courtine et al. reported that in a rat model of complete spinal cord injury, the combination of treadmill training, serotonergic agonists, and epidural electrical stimulation enabled weight-bearing treadmill locomotion [14]. However, treadmill-based gait training is performed during the chronic phase after spinal cord injury and is difficult to perform in the acute phase.

Jung et al. previously created electric stimulation models using rats as a model of functional NMES, in which they stimulated the motor points of the agonist muscles of the limbs using embedded electrodes. They also conducted a basic experiment to evaluate the stimulation conditions required to elicit synkinesia in each joint during gait [15–17]. As a result, they reported significant short-term improvements in the hindlimb synkinesia of rats with incomplete spinal cord injuries after NMES therapy [18]. NMES therapy can be used in the acute stage post-injury; however, in this model, electrodes are embedded, making it a highly invasive procedure. Therefore, a less invasive model is required to examine the effect of combined therapy in spinal regeneration.

Low-frequency pulsed currents (LPCs) and kilohertz-frequency alternating currents (KACs) are used to clinically augment muscle contractions. Treatment effectiveness may be enhanced by selecting stimulation parameters that evoke the strongest contractions with minimal discomfort and fatigue. Previous research findings regarding the muscle fatigue and degree of discomfort associated with KAC and LPC in patients with spinal cord injury are inconsistent [19, 20]. There is no report that investigates the effectiveness of NMES in stimulating gait rhythm using alternating currents in the kHz frequency for motor therapy.

We previously created a less invasive NMES therapy model using percutaneous needle electrodes and reported that kHz stimulation could be used for gait rhythm stimulation [21]. However, it is unclear whether NMES to stimulate gait rhythm using stimulation with alternating currents in the kHz frequency effectively improves motor function.

The purpose of the present study was to examine the effectiveness of using stimulation with alternating currents in the kHz frequency in improving motor function and to find the most effective NMES parameters available for gait rhythm stimulation after spinal cord injury.

## Methods

### Animals and study groups

This study was approved by the Committee for the Care and Use of Animals, Yamaguchi University (Yamaguchi, Japan). The protocols were designed and the study was conducted in accordance with the following regulations: Yamaguchi University Animal Regulations, laws on the care and maintenance of laboratory animals (Act no. 105); standards for the care and maintenance of experimental animals for the reduction of their suffering (Ministry of the Environment Public Notice no. 88); and basic policies on the use of animal experimentation at research institutions (Ministry of Education, Culture, Science, Sports and Technology Public Notice No. 7).

Twenty 12-week-old female rats (strain F334; Barcelona, Spain) weighing an average of 167 g (160–175 g) were used in this study. Thoracic spinal contusion injuries were induced in 15 rats, which were subsequently assigned to one of the following two groups: hindlimb movement therapy using NMES (iSCI-NMES, n = 10) or no treatment (iSCI-NT, n = 5). The NMES group was further divided into two groups on the basis of either a stimulation frequency of 75 Hz (iSCI-NMES 75 Hz, n = 5) or 8 kHz (iSCI-NMES 8 kHz, n = 5). Five rats that did not undergo training and in whom spinal cord injury was not induced were used as normal controls (Normal-CT, n = 5).

### Creation of incomplete spinal cord injury

Rats were anesthetized with ketamine (25 mg) and xylazine (1 mg). A midline 2-cm incision was made to expose the spinal column at the 9th thoracic spine (T9) level, and the paravertebral muscles were bilaterally dissected to visualize the transverse apophysis. Laminectomy was carefully performed at the T9 vertebral arch without damaging the facets. A 150-kdyn contusion was induced in the rats using the Infinite Horizon spinal cord injury device (model IH-400^®^, Precision Systems and Instrumentation, LLC, Lexington, KY, USA). The surgical procedure was performed using a surgical microscope (Carl Zeiss AG, Jana, Germany). The wound was subsequently irrigated with saline solution, and the muscle, fascia, and skin were reapproximated. Post-surgery, 10 mL of 0.9 % sodium chloride and 10 mg of piperacillin sodium were subcutaneously injected. Access to food and water was facilitated in custom-built cages. Post-surgical care included manual bladder expression two times daily until bladder function resumed, as well as 10 mL injections of 0.9 % sodium chloride and 10 mg piperacillin sodium two times daily for 5 days.

### NMES therapy

**Fig. 1:**
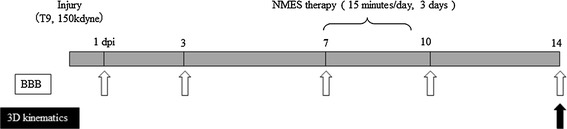
Experimental timeline. A 150-kdyn contusion was induced in the rats using the Infinite Horizon spinal cord injury device at the 9th thoracic spine (T9) level. Neuromuscular electrical stimulation (NMES) therapy (15 min/day) was administered for 3 days from the 7th day post-injury. The Basso, Beattie, Bresnahan (BBB) locomotor rating scale was performed at 1, 3, 7, 10, and 14 days post-injury (dpi). A three-dimensional (3D) kinematic analysis was performed at day 14 dpi

NMES was administered for 3 days from the 7th day post-injury (Fig. 1). Each rat was anesthetized and secured to a custom-made platform before stimulatory electrodes were percutaneously inserted into the tibialis anterior muscle and gastrocnemius muscle of both hindlimbs. To insert the electrodes near each motor point, anatomical motor point positions were referenced at the point of greatest contraction during post-percutaneous stimulation of the target muscle, using two different frequency currents (frequency, 75 Hz or 8 kHz; pulse width, 40 μs; amplitude, 1 mA; duration, 200 ms) [21].

Immediately after insertion of the stimulatory electrodes, strength–duration (SD) curves were generated for all muscles being studied [21] to assess whether the electrodes were positioned at appropriate target motor points. SD curves were constructed to show the relationship between the minimum current necessary to establish a stimulation threshold and pulse width. In this study, the minimum current necessary to cause visual muscle twitching was plotted by pulse widths of 20, 40, 60, 100, 200, and 500 μs per phase. The rheobase (the threshold twitch current over a prolonged pulse duration of 500 μs) and range of chronaxie (electrical current stimulus at the point where the threshold twitch current is twice the strength of the rheobase current and stimulates a muscle or neuron) were defined to assess the excitability of the muscle from the SD curves. Low rheobase and chronaxie values indicate that the electrode is positioned close to the motor point.

**Fig. 2:**
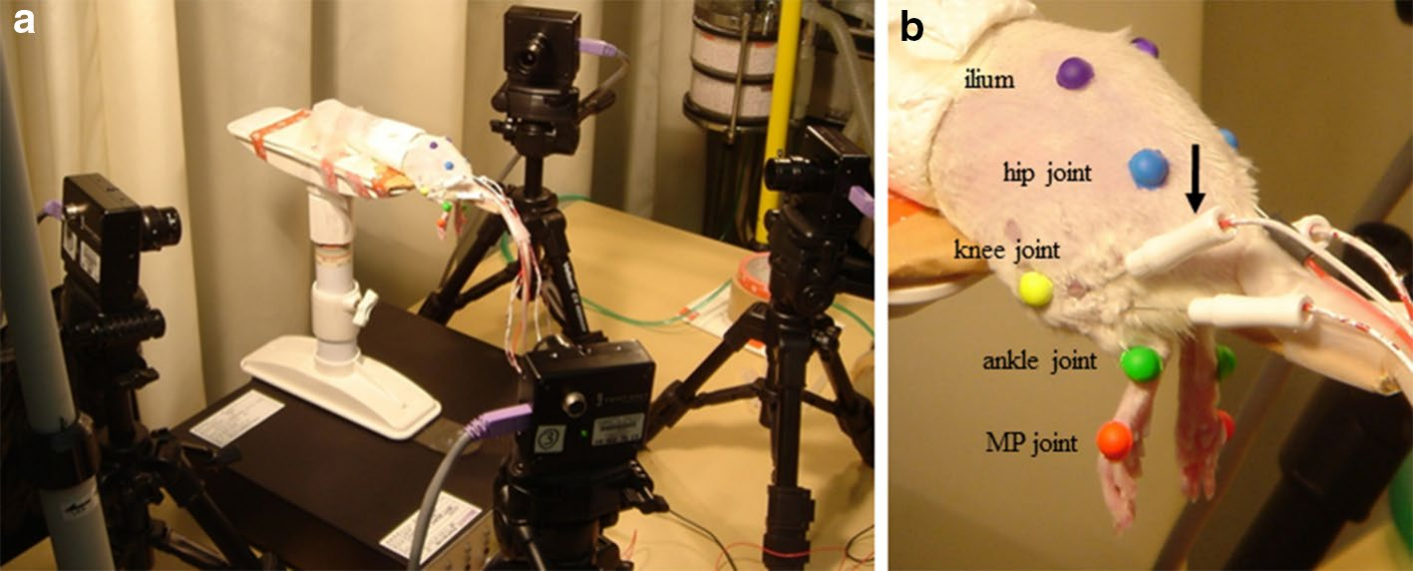
Insertion of electrodes and setting of gait rhythm stimulation. Each rat was anesthetized and secured to a platform (**a**). Needle electrodes (*down arrow*) were percutaneously inserted into the tibialis anterior (TA) muscle and gastrocnemius (Gc) of both limbs (**b**). With the rat awake and the trunk secured, gait rhythm was stimulated using stimulation electrodes and filmed using four charge-coupled device cameras (**a**)

An isolated four-channel stimulator (STG2004^®^, Multi-Channel Systems, Cytocentrics, Reutlingen, Germany) was used in this study. The stimulation parameters were calibrated to those used in previous studies [15, 21]. The amplitude of the stimulation current was set at 1.5 times the threshold known to produce visually observable twitches. An automated four-channel patch clamp system, at a pulse width of 40 μs, was used. The timing of the muscle stimulations was matched to the walking rhythm of the right and left ankle agonists when a normal rat was walking on a treadmill [22]. Stimulations were performed for 15 min while the rats were conscious. To evaluate sequential changes in the articular range of motion (ROM) of the stimulated ankle joints, we performed three-dimensional (3D) kinematic analysis using the KinemaTracer^®^ (Kissei Comtec Co., Ltd., Nagano, Japan). Color markers were attached to the joints of both hindlimbs (on the surface of the skin at the iliac region, hip, knee, ankle, and metacarpophalangeal joints). Four charge-coupled device video camera units were used to film these markers. Using kinematic analysis software, ROM was calculated and sequential changes were immediately evaluated at 5, 10, and 15 min post-stimulation (Fig. 2).

### Assessment of locomotor coordination recovery

#### Overground walking locomotor score

Locomotion was assessed through open-field locomotor testing using the Basso, Beattie, Bresnahan (BBB) locomotor rating scale; this was performed at 1, 3, 7, 10, and 14 days post-injury (Fig. 1).

#### Hindlimb coordination during treadmill walking via kinematic analysis

Three-dimensional kinematic measurements, during treadmill walking 14 days post-injury (Fig. 1), were used to characterize intra- and interlimb coordination [18]. The analysis provided joint angle trajectories during the stance and swing phases as well as the touchdown (TD) and lift-off (LO) events during multiple gait cycles.

**Fig. 3:**
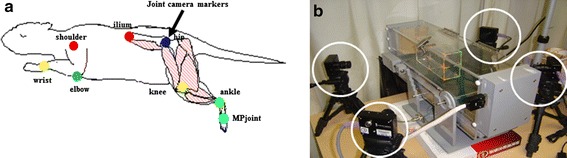
Three-dimensional gait analysis. Camera markers were attached to each joint of the four limbs, and the rat was made to walk at a set speed on the treadmill. The camera markers (*down arrow*) (**b**), which were attached to the lower limbs, were filmed using four charge-coupled device cameras (*unfilled circle*) (**a**), and the digital data obtained was analyzed and evaluated

Rats were anesthetized with 2 % sevofrane. Hair was clipped around both the upper and lower limbs, and skin glue markers were placed over 16 anatomic landmarks on the bilateral side of the shoulder, elbow, wrist, iliac crest, greater trochanter, knee joint, lateral malleolus, and fifth metatarsal head. Hemispherical markers were prepared using 6-mm doll eyes that were air painted in blue, orange, red, green, and yellow, and then coated with bitter spices to keep the test rats from biting them. One operator performed all marker placements to avoid inter-tester variability. All rats were made to walk on a treadmill (MELQUEST Co.: TMC-100) at 13.7 cm/s, and results were recorded and analyzed using the Kinema Tracer^®^ (Kissei Comtec Co., Ltd., Nagano, Japan), which was equipped with four high-speed digital cameras at 120 fps and controlled using IEEE1394 cables (Fig. 3). Kinematic data were collected at a sampling rate of 1000 Hz. This system can analyze stick pictures, time–distance factors, joint angles, joint acceleration, and Lissajous figures. The four cameras were positioned right and left anteriorly and right and left posteriorly. We used an original clear box (56 × 106 × 206 mm) for camera calibration that contained 18 calibration points with controls (corners and middles). As previously noted, rats were made to walk at treadmill speeds of 13.7 cm/s. Rotations per minute on the treadmill were calculated at 0.273 cm/s; thus, 50 rpm equaled 13.7 cm/s. The images were recorded for 11 s, which is the maximum time allowed by the system.

The video frame, in which the hindlimb toes of a rat touched the treadmill belt, was recorded as a touchdown (TD) event. Similarly, a liftoff (LO) event was marked when a rat’s hindlimb toes lifted off the treadmill belt. This approach for marking the TD and LO events has been used to describe gait kinematics in a previous report [22]. A step cycle was considered to be the duration from right TD to right TD. Each cycle was normalized to 100 % and calculated on a cycle-by-cycle basis.

Normalized joint angle trajectories and joint angle–angle plots for right and left ankle movements were used to assess intra- and interlimb coordination. Right–left symmetry during movement was quantitatively analyzed using the interlimb angle–angle plots [22]. Each normalized cycle is represented by 201 data points. A right ankle joint angle at any given point within a cycle, i (θRAi), can be used to predict the left ankle joint angle at i + 101 (θLAi + 101) for all points in the data set (the first 100 points from the first cycle of the right joint and the last 100 points from the last cycle of the left joint were discarded). The difference between the points and the line was then calculated to determine the symmetry error. The root mean square (RMS) error was calculated to characterize the entire normalized dataset with a symmetry index as follows:

$$ SYM\_E = \sqrt {\frac{{\sum\nolimits_{n} {\left( {\frac{{\theta RH_{i} - \theta LH_{i + 101} }}{\sqrt 2 }} \right)^{2} } }}{n}} $$

**Fig. 4:**
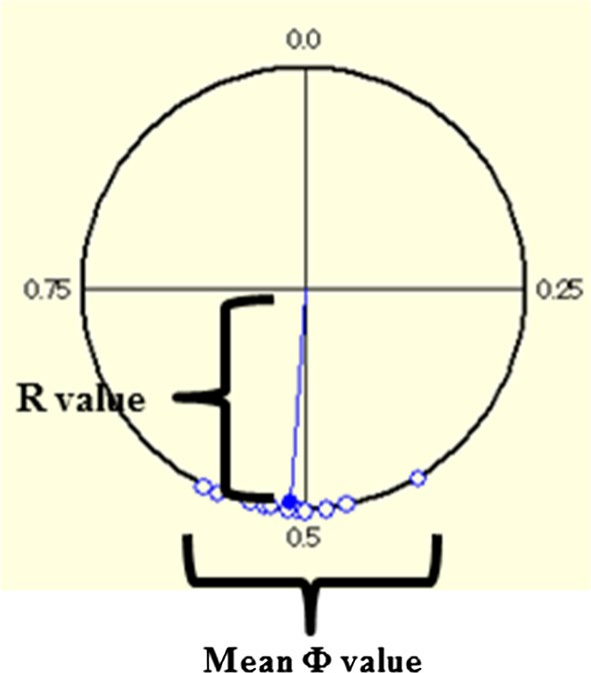
Circular phase. The touch-down phases of both hindlimbs were quantitatively assessed within one cycle as the time taken from when the right hindlimb lifted-off till the time it touched-down again. During this, the touch-down timing of the left hindlimb was plotted on a pie chart, which provided two indicators: the R and mean Φ values. The mean Φ value was normalized to 50 %. Some dispersion was shown in the R values, with dispersion decreasing as the R value approached 1 and increasing as the R value approached 0. That is, when the mean Φ value was normalized to 0.5, the R value approached 1

Quantitative assessment of interlimb gait coordination was conducted to determine consistency of 1:1 correspondence between the right and left hindlimbs and to establish the relative phase of each limb TD with respect to another within a gait cycle. In this study, mean Φ and R values were calculated in a circular phase to assess the consistency of 1:1 hindlimb right–left interlimb coordination (Fig. 4). The mean Φ values were normalized to 0.5. Some dispersion was shown by the R values, with dispersion decreasing as the R value approached 1 and increasing as the R value approached 0. The mean Φ and R values were compared among the iSCI-NMES 75 Hz, iSCI-NMES 8 kHz, iSCI-NT, and Normal-CT groups.

### Statistical analysis

To analyze BBB scores, the Welch test was performed with the independent variables between the groups. To analyze 3D kinematics data during walking at day 14 post-injury, the Welch test was used to compare the phase values of gait cycles and SYM-E values for the ankle joint ROM between the groups. In this study, a p value of <0.05 was considered statistically significant.

## Results

### Ankle-joint angle movement during NMES

**Fig. 5:**
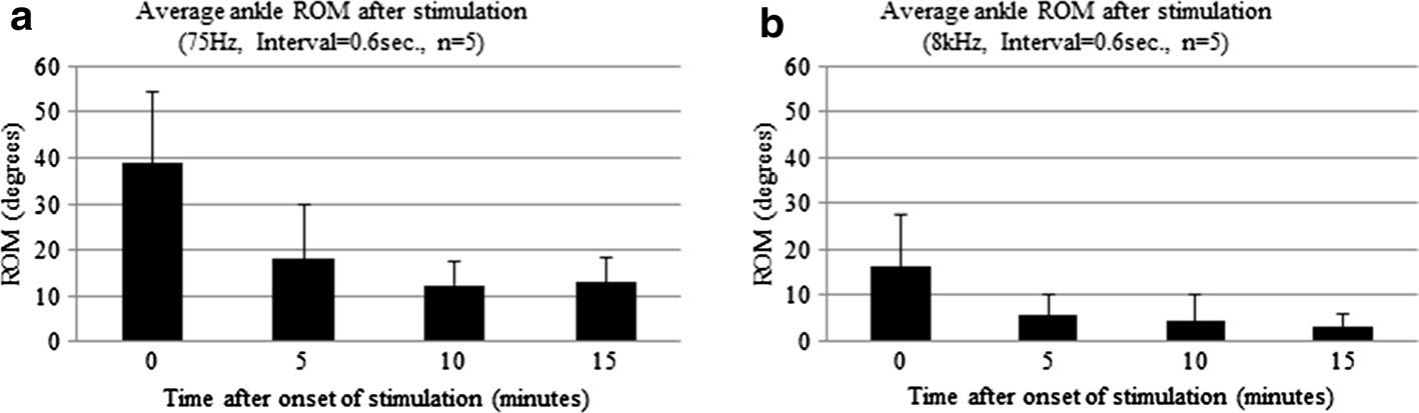
Changes in mean leg range of motion over time. The changes in mean limb joint range of motion (ROM) immediately after the start of stimulation and at 5, 10, and 15 min later. **a** In the 75-Hz stimulation frequency group, good ROM is observed immediately after the start of stimulation; however, a significant decrease in ROM is observed 5 min later (p = 0.001). **b** In the 8-kHz stimulation frequency group, joint ROM is lower than that of the 75-Hz group immediately after stimulation, and then significantly decreases after 5 min

Figure 5 shows the average ankle joint ROM immediately after stimulation and at 5, 10, and 15 min post-stimulation. During the first 10 cycles of stimulation, the average observed ankle ROM of the iSCI-NMES 75-Hz group was 39°. After 5 min of NMES, the range decayed to 18 degrees, representing a significant decrease (Fig. 5a). The average observed ankle ROM of the iSCI-NMES 8-kHz group was 16 degrees. After 5 min of NMES, the range decayed to 6°, representing a significant decrease. The average ankle ROM of both groups remained greater than zero until the completion of the NMES session; however, significant decay continued at 10 and 15 min post-stimulation (Fig. 5b).

### Over-ground walking locomotor score

**Fig. 6:**
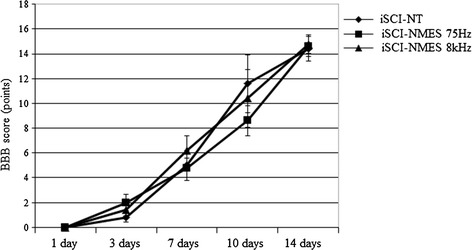
BBB score. There is no significant difference in *BBB scores* observed between either experimental group at any time

The mean BBB scores of the iSCI-NT group were as follows: 1 day post-injury, 0 ± 0; 3 days post-injury, 0.8 ± 0.4; 7 days post-injury, 5.0 ± 1.2; 10 days post-injury, 11.6 ± 2.3; and 14 days post-injury, 14.4 ± 1.0. The mean BBB scores of the iSCI-NMES 75-Hz group were as follows: 1 day post-injury, 0 ± 0; 3 days post-injury, 1.4 ± 0.5; 7 days post-injury, 6.2 ± 1.2; 10 days post-injury, 10.4 ± 2.3; and 14 days post-injury, 14.8 ± 0.7. Moreover, the mean BBB scores of the iSCI-NMES 8-kHz group were as follows: 1 day post-injury, 0 ± 0; 3 days post-injury, 1.4 ± 0.5; 7 days post-injury, 6.2 ± 1.2; 10 days post-injury, 10.4 ± 2.3; and 14 days post-injury, 14.8 ± 0.7. There were no significant differences in BBB scores between the groups (Fig. 6).

### Recovery of locomotor coordination in 3D kinematic analysis

#### Phase relationships in the gait cycle (Fig. 7)

**Fig. 7:**
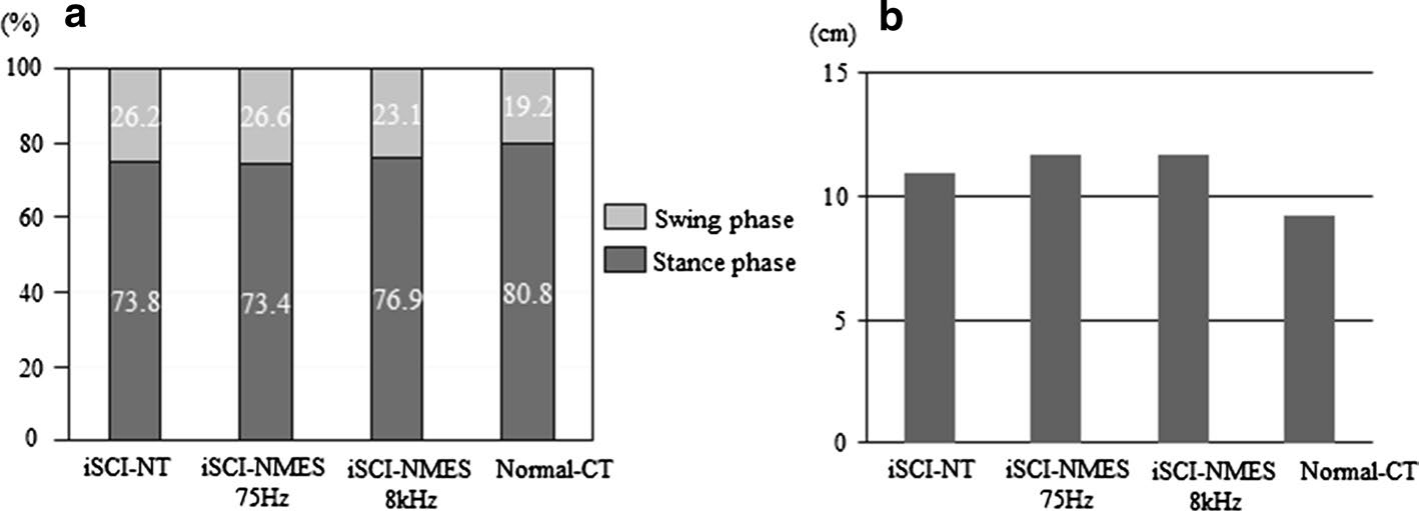
Phase relationships in gait cycle. These graphs show the two phases for stance and swing (**a**), stride length (**b**). There were no significant differences between the iSCI-NMES and iSCI-NT groups

In the Normal-CT group, the stance phase value was 80.8 ± 2.9 %, the swing phase value was 19.2 ± 2.9 %, and the double support phase value was 30.4 ± 9.4 % at 13.7 cm/s. Stride length value was 9.3 ± 4.4 cm. For the iSCI-NT group, the stance phase value was 73.8 ± 7.8 %, the swing phase value was 26.2 ± 7.8, and the double support phase value was 20.8 ± 16.9 %. The stride width value was 10.4 ± 4.8 cm. For the iSCI-NMES 75-Hz group, the stance phase value was 73.4 ± 6.2 %, the swing phase value was 26.6 ± 6.2 %, the double support phase value was 16.0 ± 10.8 %, and the stride length value was 11.7 ± 1.8 cm. For the iSCI-NMES 8-kHz group, the stance phase value was 76.9 ± 5.3 %, the swing phase value was 23.1 ± 5.3 %, the double support phase value was 35.8 ± 12.0 %, and the stride length value was 11.6 ± 1.5 cm. The injury group (iSCI-NT), treatment groups (iSCI-NMES 75 Hz and 8 kHz), and normal group (Normal-CT) showed no significant difference in the ratio of the two phases for stance and swing (Fig. 7a). Furthermore, compared with the normal group, the injury group and treatment group tend to exhibit longer stride length (Fig. 7b); however, there is no significant difference observed for either.

#### Foot trajectories and normalized joint angles

**Fig. 8:**
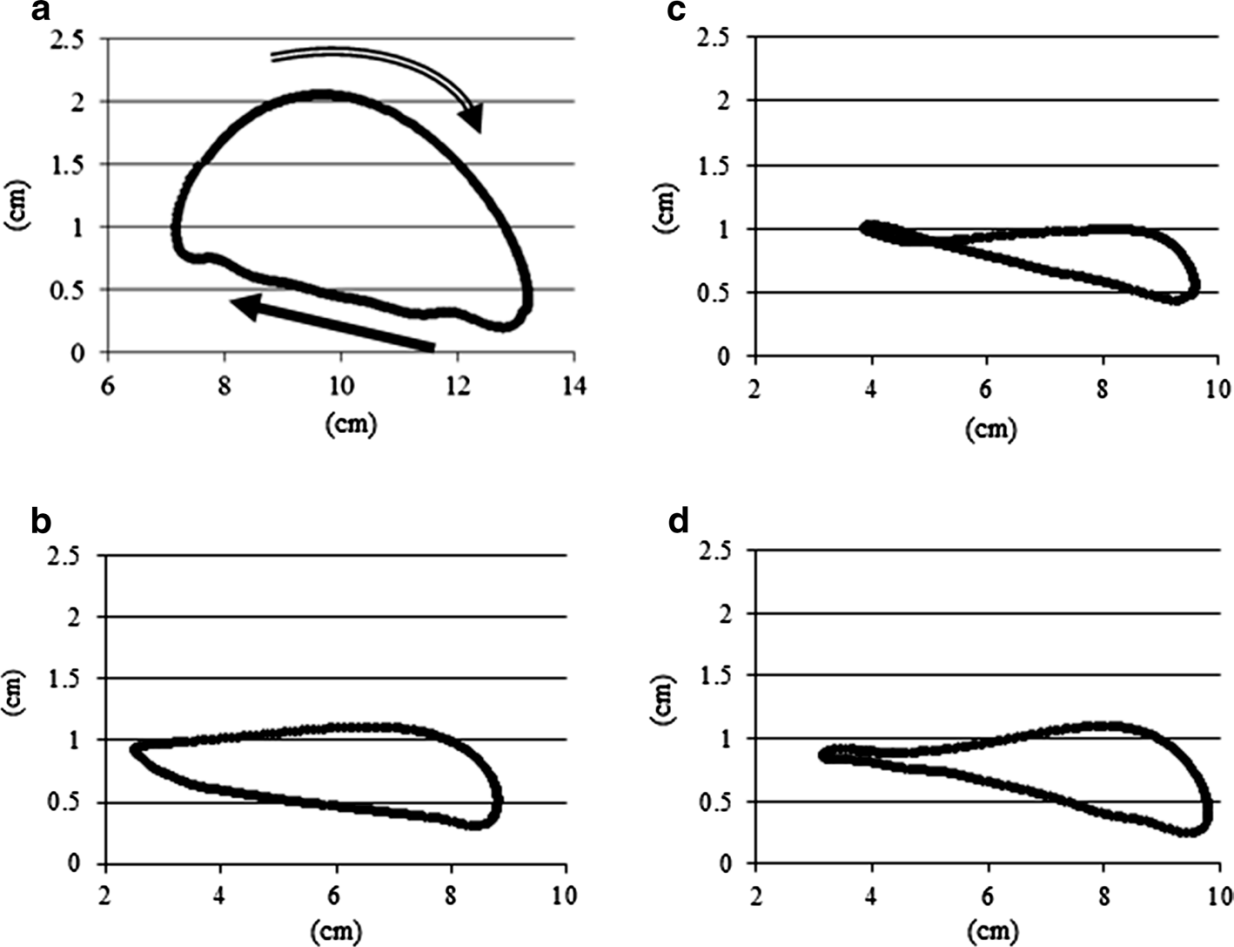
Foot trajectories. This graph shows the sagittal trajectory of the leg markers. In the normal group, the leg moves *linearly vertically* during the stance phase (*filled right arrow*); then, during the swing phase (*right double arrow*), swings up *vertically* in an arc and swings forward (**a**). In the spinal injury control group, the *vertical* movement during the stance phase is not linear (**b**); however, in the NMES treatment group, all movements are linear and an improvement is observed (**c** 75 Hz, **d** 8 kHz). On the other hand, during the swing phase, compared with the normal group, no groups exhibited *vertical arc-like* movements and there was no improvement in *horizontal* leg movement during the swing phase

The graph marking the sagittal trajectory of the leg markers shows that in the normal group, the leg moves linearly vertical during the stance phase, then swings up vertically in an arc, and swings forward (Fig. 8a). In the spinal injury control group, the vertical movement during the stance phase was not linear (Fig. 8b); however, in the NMES treatment group, all movements were linear and an improvement was observed (Fig. 8c, d). On the other hand, during the swing phase, compared with the normal group, none of the experimental groups exhibited vertical arc-like movement; there was no improvement in horizontal leg movement during the swing phase (Fig. 8).

**Fig. 9:**
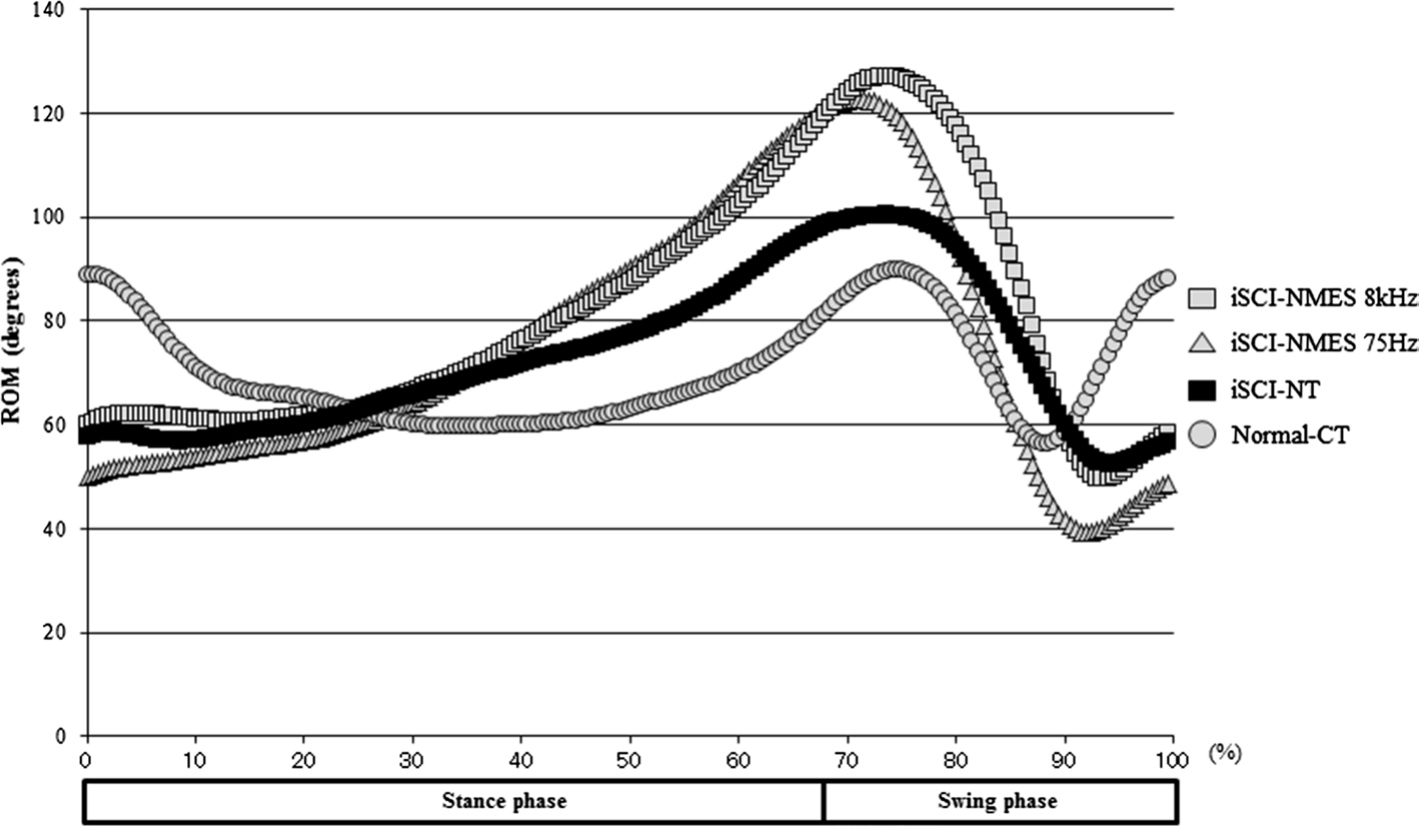
Normalized joint angles (three-directional mean graph). This graph shows the normalized range of motion trajectory for joints. Although joint angle range of motion changes during the swing phase in the treatment group, the change is not great compared with the injury control group

**Fig. 10:**
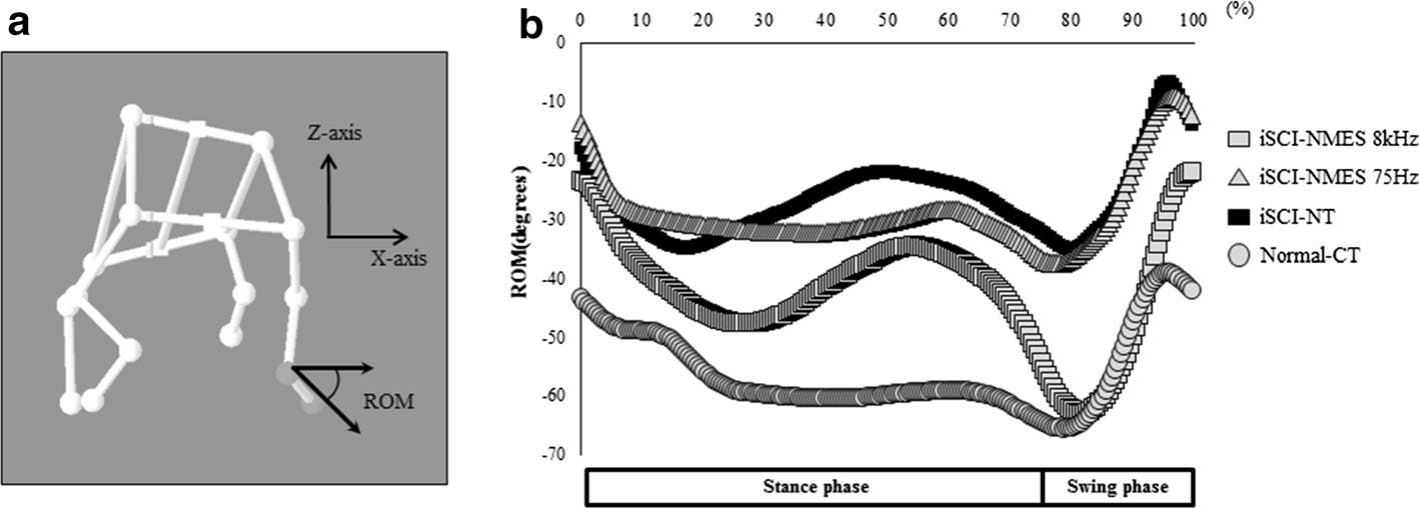
Normalized joint angles (mean graph of the coronal plane). This graph shows the joint range of motion trajectory in the coronal plane to assess ankle eversion (**a**), which becomes close to the pattern of the normal group in the 8-kHz group (iSCI-NMES 8 kHz), particularly during the swing phase (**b**), and an improvement in eversion is observed

In the normalized joint angle trajectory graph, ROM greatly changed during the swing phase in the treatment group (Fig. 9), whereas in the coronal plane of the joint ROM trajectory, particularly in the iSCI-NMES 8-kHz group, the pattern during the swing phase approached normal, and an improvement in ankle eversion was observed (Fig. 10).

#### Circular phase (Fig. 11)

**Fig. 11:**
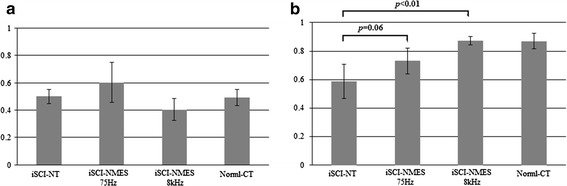
Mean Φ and R values in circular phase. The circular phase quantifies the touchdown phase of both hindlimbs. Upon comparing the circular phase of each group, there is no significant difference observed in mean Φ value (**a**); however, when comparing the experimental groups with the injury control group, the R value tends to improve in the iSCI-NMES 75-Hz group (p = 0.06), and a significant difference is observed in the iSCI-NMES 8-kHz group (p < 0.01) (**b**)

For normal rats, the mean Φ value was 0.49 ± 0.06 % and the R value was 0.87 ± 0.05. For the iSCI-NT group, the mean Φ value was 0.5 ± 0.05 and the R value was 0.59 ± 0.12. For the iSCI-NMES 75-Hz group, the mean Φ value was 0.6 ± 0.01 and the R value was 0.73 ± 0.09 (Fig. 11). For the iSCI-NMES 8-kHz group, the mean Φ value was 0.4 ± 0.08 and the R value was 0.87 ± 0.03 (Fig. 11). No significant differences were observed in the mean Φ values between the four groups. The iSCI-NT and iSCI-NMES 75-Hz groups had significantly smaller R values than the Normal-CT group; however, the iSCI-NMES 75-Hz group exhibited greater improvement than the iSCI-NT group (p = 0.06; Fig. 11). The iSCI-NMES 8-kHz group had significantly smaller R values than the iSCI-NT group (p < 0.01; Fig. 11).

#### Angle–angle plots

**Fig. 12:**
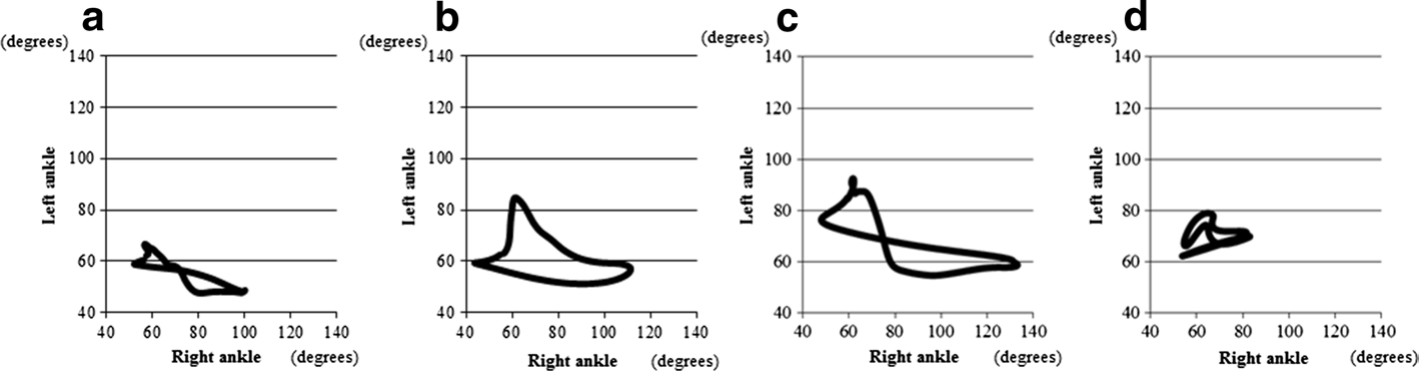
Angle–angle plots. This graph shows the angle–angle plots of the joints in each group. The symmetry index is 18.0 ± 7.9 in the Normal-CT group (**a**), 17.7 ± 9.0 in the iSCI-NMES 75-Hz group (**b**), 15.4 ± 7.4 in the iSCI-NMES 8-kHz group (**c**), and 7.6 ± 2.0 in the Normal-CT group (**d**). Although a marginal improvement is observed in the iSCI-NMES 8-kHz group, no significant improvement is achieved

An angle–angle plot graph of the left and right ankles determined by 3D kinematic gait analysis revealed that when walking at the speed of 13.7 cm/s, the symmetry index was 7.6 ± 2.0 in the Normal-CT group, 18.0 ± 7.9 in the NT group, 17.7 ± 9.0 in the iSCI-NMES 75-Hz group, and 15.4 ± 7.4 in the iSCI-NMES 8-kHz group. Although improvements were insufficient, a marginal improvement was observed in the iSCI-NMES 8-kHz group (Fig. 12).

## Discussion

Central pattern generators (CPG) in brainstem–spinal cord neural networks associated with gait, producing rhythmic movements. These stimulate peripheral sensory input from muscular spindles and the golgi tendon organ, which contribute to the mechanism in which NMES improves motor function, and this repetitive peripheral sensory input is required for neural plasticity [23, 24]. Jung et al. reported that in a NMES rat model using embedded electrodes, the stimulation of gait rhythm after incomplete spinal cord injury significantly improved motor function of the hindlimbs in the short term [18]. In a previous rat NMES model using needle electrodes, we successfully created a less-invasive NMES therapy model [21]. On the other hand, we were unable to find any reports examining conditions under which the stimulation of gait rhythm using NMES effectively improved motor function.

When stimulating gait rhythm using NMES, muscle fatigue can be a problem. In the report by Jung et al. describing embedded electrodes used in the agonist muscles of the hip, a significant decrease in ROM was observed less than 1 week after the start of stimulation [18]. In a previous NMES model that used needle electrodes in normal rats, gait rhythm was successfully stimulated under sedation; however, a significant decrease in ROM was observed less than 5 min after the start of stimulation [17]. This was attributed to fatigue of the stimulated muscle because of open-loop stimulation. To address this reduced ROM due to muscle fatigue, stimulation with a higher instead of a lower frequency is an effective method to increase the electrical stimulation current threshold and impede muscle fatigue [25]. Furthermore, Ward et al. reported that stimulation with alternating currents in kHz frequency is effective for muscle fatigue in NMES [26, 27]. We previously reported that when stimulating gait rhythm, stimulation with alternating currents in kHz frequency could possibly be used for motor therapy [17]. In the present study, ROM was significantly smaller in the 8-kHz group than in the 75-Hz group, and the effect on muscle fatigue was not fully examined. As previously reported, this is believed to be because the stimulation intensity was set at three times the threshold known to produce a visually observable twitch. However, to maintain the conditions other than the stimulation frequency in the present study, the stimulation intensity was 1.5 times the threshold intensity that produces a visually observable twitch. In a similar model using embedded electrodes in the agonist muscles of the hip, Jung et al. reported that by day 14 post-injury, NMES treatment given 15 min/day for 5 days, starting 1 week after injury, resulted in a significant improvement in the touchdown phase of both hindlimbs, as well as in the coordination of the left and right hips [18]. In the present study, we observed an improvement in the touchdown phase of both hindlimbs in the iSCI-NMES 75-Hz group, as well as a significant improvement in the iSCI-NMES 8-kHz group (Fig. 11). Although the interlimb coordination of both hindlimbs tended to improve, there was no significant improvement observed. Therefore, in Jung et al.’s model [18], since the treatment was given for 5 days and embedded electrodes were used, stimulation of gait rhythm via actual stimulation of motor points during the 15-min stimulation period may have been enabled. However, in a study with identical conditions other than the stimulation frequency, motor function improved more in the iSCI-NMES 8-kHz group than in the iSCI-NMES 75-Hz group. This result supports the notion that kHz stimulation is effective in the stimulation of gait rhythm to treat spinal injury-induced motor paralysis.

Although the mechanism in which stimulation with alternating currents in kHz frequency effectively improves motor function is not understood in detail, as reported by Ward et al., stimulation with alternating currents in kHz frequency effectively stimulates fatigue-resistant fibers and may produce greater sensory-feedback with the central pattern generator [27].

Limitations of the present study were that at 3 days, the stimulation treatment period was short and that evaluations were only conducted for 2 weeks. In future, there should be a longer period of study. Furthermore, a stimulation frequency of 8 kHz has not been shown to be clinically effective; therefore, examinations should be performed under conditions that enable the clinical application of kHz frequency stimulation. In future, we plan to examine stimulation conditions that effectively improve motor function, as well as the effect of combination therapy with various regenerative therapies to help establish effective rehabilitation during the acute phase after spinal cord injury and regenerative therapy.

## Conclusions

We employed a less-invasive NMES therapy model with needle electrodes to examine the effectiveness of high-frequency stimulation for gait rhythm. Three-dimensional gait analysis revealed improved toe clearance and touchdown phase of both hindlimbs in the NMES group, with a particularly significant improvement in the 8-kHz group. This suggests that stimulation with alternating currents in the kHz frequency is effective in gait rhythm stimulation by NMES.
